# De-Coding the Contributions of the Viral RNAs to Alphaviral Pathogenesis

**DOI:** 10.3390/pathogens10060771

**Published:** 2021-06-19

**Authors:** Autumn T. LaPointe, Kevin J. Sokoloski

**Affiliations:** 1 Department of Microbiology and Immunology, School of Medicine, University of Louisville, Louisville, KT 40202, USA; altown02@louisville.edu; 2 Center for Predictive Medicine and Emerging Infectious Diseases, University of Louisville, Louisville, KT 40202, USA

**Keywords:** alphavirus, virulence, viral pathogenesis, viral RNA

## Abstract

Alphaviruses are positive-sense RNA arboviruses that are capable of causing severe disease in otherwise healthy individuals. There are many aspects of viral infection that determine pathogenesis and major efforts regarding the identification and characterization of virulence determinants have largely focused on the roles of the nonstructural and structural proteins. Nonetheless, the viral RNAs of the alphaviruses themselves play important roles in regard to virulence and pathogenesis. In particular, many sequences and secondary structures within the viral RNAs play an important part in the development of disease and may be considered important determinants of virulence. In this review article, we summarize the known RNA-based virulence traits and host:RNA interactions that influence alphaviral pathogenesis for each of the viral RNA species produced during infection. Overall, the viral RNAs produced during infection are important contributors to alphaviral pathogenesis and more research is needed to fully understand how each RNA species impacts the host response to infection as well as the development of disease.

## 1. Background

Alphaviruses are single-stranded, positive-sense RNA viruses that are naturally transmitted between a mosquito vector and a vertebrate host. Epizootic spillover events result in the infection of humans and horses, potentially resulting in severe disease. Alphaviruses are largely classified as either arthritogenic or encephalitic based on the symptoms of infection. Arthritogenic alphaviral infection causes disease varying from mild to severe multi-joint arthritis and can persist for several months to years past the acute phase of infection [1]. This includes Chikungunya (CHIKV) and Ross River Virus (RRV) which are capable of causing debilitating polyarthritis as well as the model alphavirus Sindbis virus (SINV), which is the causative agent of rash-arthritic diseases like Pogosta disease, Ockelbo disease, and Karelian fever [2,3,4,5]. While not associated with high rates of mortality, the high morbidity of arthritogenic alphaviral disease results in a high economic burden that is particularly damaging in regions where labor-intensive work is prevalent [6,7]. The encephalitic alphaviruses include Venezuelan, Eastern, and Western Equine Encephalitis viruses (VEEV, EEEV, and WEEV) and are capable of causing severe meningitis and encephalitis, as well as long-lasting sequelae such as seizures, paralysis, and cognitive deficits in survivors [8,9,10,11]. These viruses, while comparatively rare in regards to their incidence, typically have high mortality rates, especially in comparison to the arthritogenic alphaviruses, with viruses like EEEV having mortality rates as high as 70% in symptomatic individuals [12]. Despite the threat that alphaviruses pose to public health, there are no antiviral strategies or vaccines for preventing alphaviral infection or treating alphaviral disease. This deficit of viable therapeutics highlights the need to better understand the mechanisms behind alphaviral infection and pathogenesis in order to develop novel antiviral strategies for the mitigation of alphaviral disease.

## 2. The Contribution of Alphaviral RNAs to Pathogenesis, an Emerging Frontier

**Figure 1:**
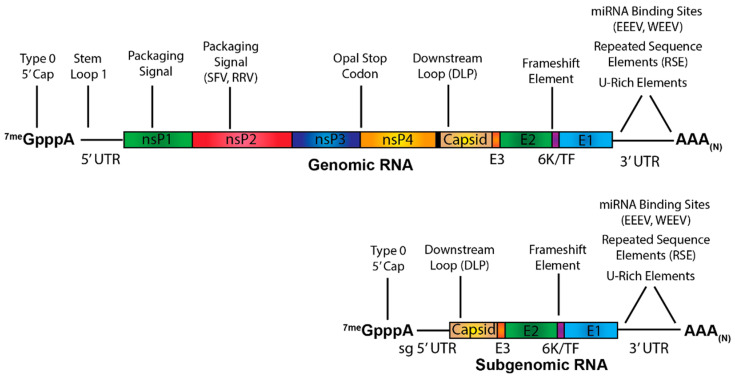
Alphaviral Genetic Organization and RNA Virulence Traits. Sequences and secondary RNA structures in the genomic and subgenomic RNA known to impact virulence are indicated. Elements that have only been shown to be present in a subset of alphaviruses have the specific viruses listed in parentheses. sg 5′UTR = subgenomic 5′ UTR.

The primary purpose of this review is to summarize what is known about how the viral RNAs (vRNAs) produced during alphaviral infection influence pathogenesis (Figure 1). While much has been written about the role of viral RNA in translation, RNA synthesis, and overall viral replication, relatively little is known about how the viral RNA influences alphaviral pathogenesis, and what information does exist on the topic has never been formally summarized, leaving the current state of the field scattered. Thus, the purpose of this review is to amass what is known about alphaviral RNA virulence traits and identify what is still unknown regarding the contribution of the viral RNA to alphaviral pathogenesis.

## 3. Alphaviral RNA Species and Viral Replication/Transcription

**Figure 2:**
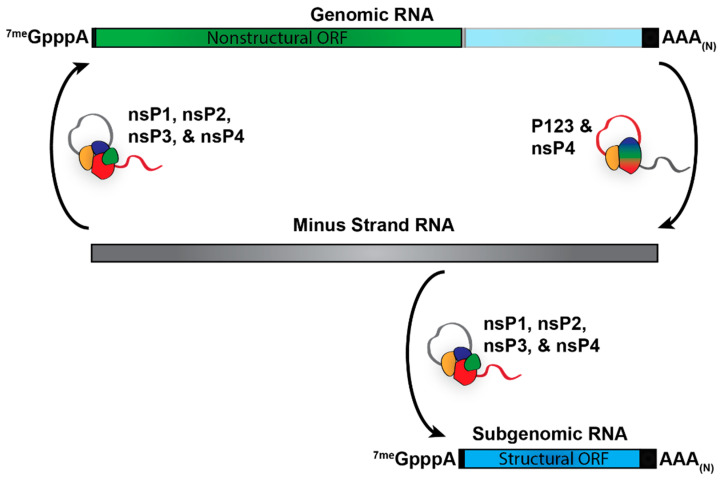
The Products of Alphaviral RNA Replication. Translation of the viral genome produces the nonstructural polyprotein P1234. nsP4 is first cleaved off and associated with P123 in order to form the complex that is responsible for synthesizing the minus-strand RNA template. P123 is then further processed into the individual nonstructural proteins nsP1, nsP2, and nsP3. The four fully processed nonstructural proteins then form the replication complex responsible for synthesizing the genomic and subgenomic RNA.

Alphaviruses produce primarily three RNA species during viral replication: the genome, which encodes the nonstructural proteins; the minus-strand RNA, which serves as the template for viral replication and transcription; and the subgenome, which encodes the structural proteins (Figure 2). The specific synthesis of the RNA species is in part determined by the processing of the nonstructural polyprotein and the promoter sequences present on the viral RNAs. Translation of the genomic RNA produces the nonstructural polyprotein P1234. To form the initial viral replicase complex, nsP4 is cleaved from the polyprotein by the proteolytic activities of nsP2 and associates with P123 [13]. This initial complex then uses the viral genome as a template for the synthesis of the negative-sense minus-strand RNA. The nonstructural polyprotein is then further cleaved by nsP2 to produce the individual nonstructural proteins, nsP1-4 that presumably in conjunction with host factors, form the fully processed viral replicase complex. Once fully processed, the replicase complex then uses the minus-strand RNA as a template to synthesize the genomic and subgenomic vRNAs. For an in-depth discussion of alphaviral replication, see Rupp et al., 2015 [14].

## 4. Contributions of the Alphaviral Positive-Sense RNAs to Pathogenesis

### 4.1. The Contribution of Non-Templated Features of Alphaviral RNAs

From a compositional perspective, there are two features of the alphaviral RNAs that arise in a non-templated fashion during replication/transcription. These two features are the 5′ cap structure and the 3′ poly(A) tail. Despite being non-templated, these RNA features arise from the specific activities of the alphaviral replication complex; with the addition of the 5′ cap being due to the concerted activities of nsP1 and nsP2, and the addition of the poly(A) tail is due to nsP4. While, to date, there have been no reported virulence determinants associated with the 3′ poly(A) tail, the 5′ cap structure, in contrast, has been associated with virulence at several levels.

The alphaviral cap structure is different from host cap moieties, creating a major means by which the host is able to distinguish between self and viral RNAs. More specifically, eukaryotes incorporate a Type-1 cap that is 2′O methylated [15]. Cytosolic RNAs lacking a Type-1 cap structure are susceptible to detection by a number of host proteins, leading to the activation of host antiviral responses. In contrast, alphaviruses incorporate a Type-0 cap structure, which is similar to the Type-1 cap with the exception that the adjacent nucleotide to the 7-methyl guanosine cap residue is not 2′O methylated. Regardless of the differences between the viral Type-0 cap and the host Type-1 cap structures, the presence of the cap serves to functionalize the viral RNAs as mRNAs and to protect from sensing of the 5′ terminus from cytosolic RNA helicase sensors such as RIG-I and MDA5 [16].

Nonetheless, the alphaviral capping machinery is not absolutely efficient. Recent work from our lab has demonstrated that a significant proportion of the genomic RNAs produced during alphaviral infection lack a 5′ cap structure [17]. In lieu of the Type-0 cap structure, these noncapped genomic RNAs predominantly have 5′ monophosphate termini. Nonetheless, even though the vast majority of the noncapped genomic RNAs have a 5′ monophosphate rendering them undetectable by RIG-I, a considerable portion contain a 5′ di- or tri-phosphate terminus which could be potentially be detected by RIG-I [18].

While the direct mechanistic impact of the noncapped genomic RNAs remains a topic of ongoing assessment, altering the ratio of capped/noncapped genomic vRNA produced during infection is known to have significant impacts on alphaviral pathogenesis. More specifically, increasing the proportion of capped genomic RNA produced during SINV infection of 4-week old C57BL/6 mice resulted in almost complete ablation of morbidity and mortality as well as significantly reduced levels of inflammatory cytokine transcripts compared to wild type SINV infection [19]. Importantly, while morbidity and mortality were significantly affected, the increased capping mutant did not exhibit any deficits in viral replication or tropism in animal models of infection. These findings suggest that the noncapped genomic RNA produced by SINV may therefore be a primary inducer of pathogenesis. While the mechanism as to how the noncapped genomic vRNA modulates inflammation and disease has not yet been characterized, this study does show that the presence or absence of the 5′ cap structure on the viral genomic RNA is an important determinant of alphaviral pathogenesis.

### 4.2. The Contribution of Untranslated Regions to Alphaviral Pathogenesis

Due to the genetic arrangement of the alphaviruses, the genomic and subgenomic viral RNAs possess shared and nonshared untranslated regions. Specifically, the 5′ untranslated regions (UTRs) of the genomic and subgenomic RNAs are different from one another due to the utilization of an internal initiation site on the minus strand template RNA for subgenomic RNA synthesis, whereas synthesis of the genomic RNA begins at the 3′ terminus of the template minus-strand RNA [20]. In contrast to the 5′ UTR, the canonical 3′ UTR is conserved across the two positive-sense viral RNA species; however, the functional 3′ UTR of the genomic RNA is greatly extended as the stop codon after the nsP4 protein is a significant distance from the stop codon of E1 and the 3′ poly (A) tail.

#### 4.2.1. The 5′ Untranslated Regions and Adjacent Sequences

The alphaviral 5′ UTR varies in both length and sequence among the different alphaviruses and is typically between 27 and 85 nucleotides long [21]. Although there is great variety in the 5′ UTR, there are several features that are prevalent across the members of the alphaviral genus. These include a number of structural RNA elements which act as cis-acting features to direct viral RNA synthesis. On the genomic RNA, these structural elements include the 5′ conserved sequence element (CSE), a 51nt stem-loop structure necessary for the initiation of viral RNA synthesis [22]. The complementary sequence/structure found in the context of the minus strand template RNA is also functionally important for viral replication. In close proximity to the 5′ CSE is the second set of stem-loop structures, which despite being in the coding frame of the nsP1 gene, they are considered part of the replication cis-acting RNA elements due to their capacity to enhance the transcriptional activity from the 5′ CSE [22]. As these elements direct viral RNA synthesis they are undoubtedly involved in pathogenesis; however, as these elements are required for alphaviral RNA replication they are not considered specific virulence traits.

**Figure 3:**
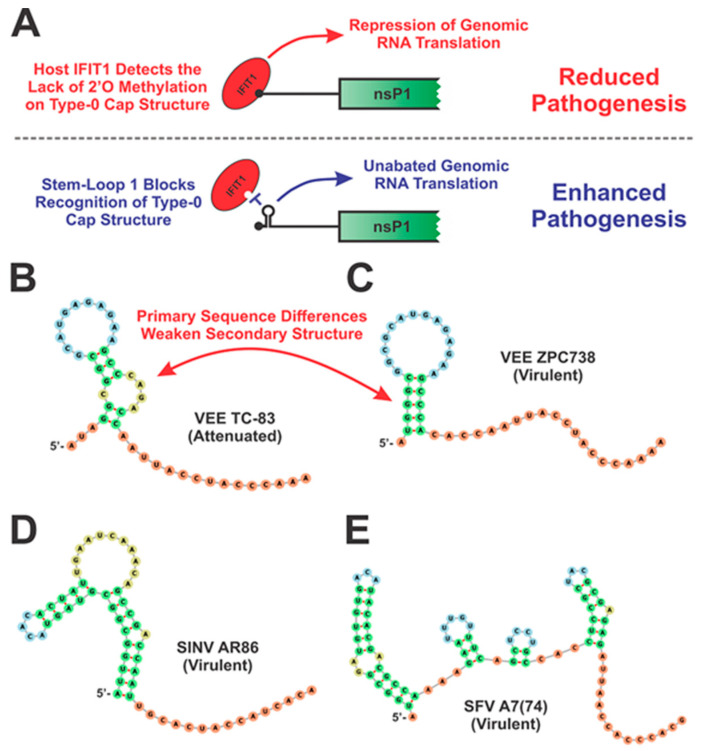
Predicted Secondary Structures of Alphavirus 5′ UTRs. (**A**) IFIT1 recognizes non-self RNA partially through the absence of 2′O methylation on the 5′ cap structure. In the absence of 2′O methylation, IFIT1 binds to the 5′ end of the RNA and represses viral translation, leading to reduced pathogenesis (**A**, top). The 5′ stem-loop structure in the alphaviral 5′ UTR blocks binding of IFIT1 to the viral RNA, preventing translational inhibition and host detection, leading to enhanced viral pathogenesis (**A**, bottom). The stem-loops in the 5′ UTR of VEEV TC-83 (L01443) (**B**), VEEV ZPC738 (AF100566) (**C**), SINV (U38305) (**D**), and SFV (Y17207) (**E**) [26,29,30,31]. In these structures the 5′ end is annotated and the Start codon immediately follows last nucleotide illustrated.

Nonetheless, the 5′UTR of the alphavirus genomic RNA does have structural elements which are directly involved in virulence, and the importance of these features has been demonstrated in multiple alphaviruses through point mutations of specific residues. Specifically, mutation of nucleotide 3 in VEEV has been shown to result in avirulence in immunocompetent mice but does not affect pathogenesis in IFNAR-/- mice [23,24,25]. Similarly, point mutations at nucleotides 5 and 8 in SINV and at nucleotides 21, 35, and 42 in Semliki Forest virus (SFV) have also been shown to impact neurovirulence and pathogenicity in rodents [26,27,28]. The role of secondary RNA structure in modulating pathogenesis has been best shown in VEEV, where comparison of the RNA structure in the 5′ UTRs of the virulent ZPC-738 strain with that of the attenuated TC83 strain showed that the attenuated TC83 5′ UTR possessed less canonical Watson-Crick base pairing and reduced overall secondary structure compared to the virulent ZPC-738 strain (Figure 3) [29]. Furthermore, mutation of nucleotide 3 in the 5′ UTR, which has been previously established to alter virulence in VEEV, disrupts the 5′ stem-loop structure that confers resistance to detection by IFIT1, a host protein capable of recognizing non-self RNA via the detection of non-Type 1 cap structures. The binding of IFIT1serves to prevent translation initiation through the loss of eIF4E binding. This 5′ stem-loop structure was found to prevent the entry of the viral RNA into the IFIT1 RNA-binding pocket, thereby protecting the alphaviral genome from being recognized [29]. Destabilization of the 5′ UTR stem-loop resulted in susceptibility to IFIT1 in virulent strains of VEEV, as well as increased induction of type-I interferon (IFN) in tissue culture models of infection, indicating the importance of this structure to preventing the activation of the host antiviral response [30]. Whether the previously identified mutations in SINV and SFV affect RNA structure in a similar manner as seen in VEEV has yet to be determined. Given that nucleotides 5 and 8 in SINV and nucleotide 21 in SFV are all predicted to be part of a similar 5′ stem-loop structure to that of VEEV, it is likely that mutating these particular residues would disrupt the stem-loop structure resulting in IFIT1 binding. This is highly plausible given the proximity of these structures to the terminus of the 5′ UTR (Figure 3) [26,29,31]. Nucleotides 35 and 42 in SFV, however, occur in a second, separate stem-loop downstream of the first stem-loop structure. The function of this second 5′ UTR stem-loop and whether disrupting this structure would negatively affect other alphaviruses in a similar manner to SFV is currently unknown [26].

**Figure 4:**
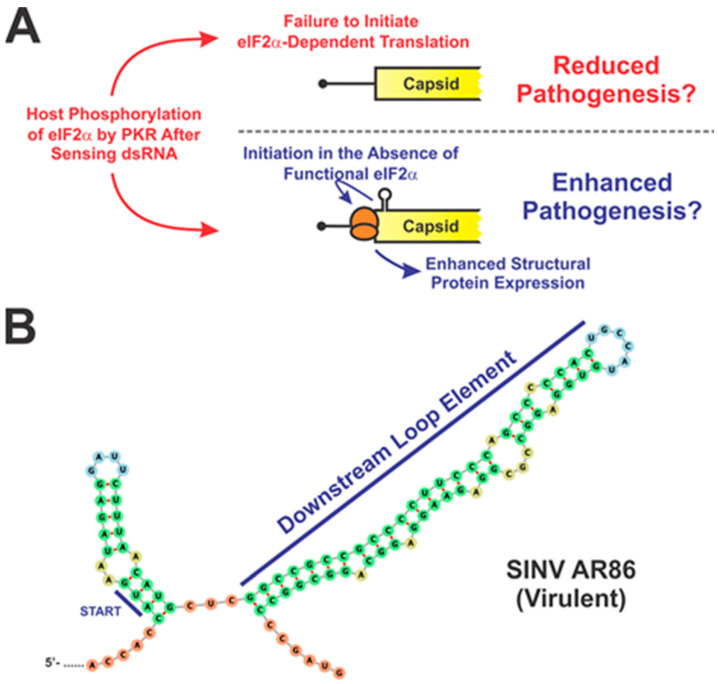
Predicted Structure and Mechanism of Action of the DLP. (**A**) During alphaviral infection, the phosphorylation of eIF2α represses translation via the disruption of translation initiation. Without the DLP, the subgenomic RNA would not be able to efficiently overcome host translational shut-off, potentially resulting in reduced viral translation and therefore reduced pathogenesis (**A**, top). The DLP acts as a translation enhancer that allows the subgenome to be translated even after host translation has been shut-off due to the phosphorylation of eIF2α by PKR. Bypassing the need for eIF2α allows for enhanced structural protein expression and serves as a potential mechanism for enhancing viral pathogenesis (**A**, bottom). (**B**) Secondary structure in the first 145nt of the subgenome of SINV AR86 (U38305) was predicted using Mfold [33]. The sequence shown starts at nt 39 of the subgenomic RNA and the structural ORF start codon is indicated.

Similar to the alphaviral genomic RNA, the 5′ UTR of the subgenomic RNA and its adjacent coding region also has a functional role in alphaviral pathogenesis through the presence of cis-acting features. However, unlike the genomic RNA, the 5′ features of the subgenomic RNA focus largely on the regulation or enhancement of translational activity. The most notable feature is the Downstream LooP (DLP), which is a stable stem-loop structure present in the subgenome located approximately 27–31nt downstream of the initiation codon of the capsid protein (Figure 4) [32]. There are two types of DLP: type A, which is larger, contains more unpaired nucleotides and is present in the SINV group; and type B, which is more compact and is present in the SFV group of alphaviruses [33]. The DLP functions as a translation enhancer allowing the subgenome to be translated even when host translation has been shut-off due to the activity of PKR, which is responsible for phosphorylating eIF2α in response to sensing dsRNA. The phosphorylation of eIF2α results in the inhibition of protein synthesis [34]. During the ribosomal scanning event associated with translational initiation, the DLP causes the small ribosomal subunit to stall in such a way that the initiation codon is lined up with the P site of the 40s ribosome [35]. This allows translation of the subgenome to be initiated without having to recruit functional eIF2α [36,37]. This is significant in the context of viral infection because PKR is highly activated during alphaviral replication [37]. By foregoing the need of eIF2α for translation initiation, the alphaviral subgenomic RNA is able to continue to be translated in spite of PKR activation, while host RNAs that are dependent on eIF2α, including those of the antiviral repsonse, are susceptible to translational shutoff. Alphaviruses mutants where the DLP has been deleted have been shown to be susceptible to translation restriction by eIF2α phosphorylation, resulting in decreased viral replication [38]. Additionally, deletion of the DLP also results in the virus being significantly more sensitive to type-I IFN, which would be detrimental to in vivo infection and pathogenesis. While the importance of the DLP to pathogenesis has yet to be directly demonstrated in an animal model of infection, these studies suggest that the DLP allows alphaviruses to evade critical aspects of the host immune response.

#### 4.2.2. The 3′ Untranslated Region

Like the 5′ UTRs, the canonical 3′ UTRs of the alphaviruses vary greatly in both sequence and length, being anywhere from ~80 to over 700 nucleotides long depending on the species [21,39]. In addition to the variable length of the 3′ UTR, the overall sequence composition and arrangement exhibits significant heterogeneity. For a comprehensive review of 3′ UTR organization, see Hyde et al., 2015 [21]. Despite the sequence heterogeneity of the alphaviral 3′UTRs, one cis-acting element- the 19nt 3′ CSE, is absolutely conserved across the genus [39]. The 3′CSE is a unique element from the 5′CSE despite being functionally related in regards to their roles in viral RNA replication. The 3′ CSE is immediately adjacent to the start of the 3′ poly(A) tail and serves as the promoter for minus-strand synthesis. As with the RNA synthetic elements of the 5′UTR, the 3′ CSE is indirectly related to pathogenesis through its role in RNA synthesis/-replication [40]. Outside of the 3′ CSE, the large amount of sequence diversity present in the 3′ UTRs of alphaviruses has made it somewhat difficult to determine functions that are conserved amongst the entire genus, but there are several aspects of the alphaviral 3′ UTRs known to be important for pathogenesis within the contexts of specific alphaviruses.

A notable element of the alphaviral 3′ UTR is the presence of Repeated Sequence Elements (RSEs) which form stem-loop structures. Although the composition, number, and length of the RSEs vary among the alphavirus genus as a whole, the RSEs of alphaviruses in the same clade retain a large degree of similarity [39]. In CHIKV, the presence of the RSEs has been positively correlated with fitness in mosquitos, but negatively correlated with fitness in mammalian models of infection [41]. In mosquito models of infection, the RSEs have been found to be required for efficient viral replication and play an important part in alphaviral transmission [42,43]. For example, duplications and sequence change the RSEs of the Asian endemic lineage of CHIKV, which diverged from the East/Central/South African (ECSA) lineage, allowed this strain to adapt to more efficient mosquito infection and transmission [21]. However, despite their importance to invertebrate infection, the RSEs are largely dispensable to replication in mammalian models of infection and their presence may actually reduce pathogenesis in vertebrates [44]. Evidence for this comes from observations that, when combined with a point mutation in the viral glycoprotein E2, the deletion of one of the RSEs in CHIKV was found to result in enhanced virulence in C57BL/6 mice [45]. Interestingly, this combination of point mutation and RSE deletion was a common adaptation found in chronically infected Rag -/- mice [45]. The precise mechanism behind how the RSEs affect CHIKV pathogenesis, and whether this is true for other alphaviruses has yet to be determined.

A second readily identifiable element of the alphaviral 3′UTR is the U-Rich Element (URE), which is typically found immediately 5′ of the 3′CSE sequence. While not strictly conserved across the genus, this element was found to interact with the host HuR protein. thereby promoting viral RNA stability via preventing transcript-specific deadenylation [46,47]. Alphaviruses lacking discernible UREs, such as RRV and CHIKV, have also been shown to interact with the host HuR protein. For these viruses, the interaction is mediated by specific RSEs which have AU nucleotide motifs [48]. Although the impact of disrupting the HuR:vRNA interaction on alphaviral pathogenesis has not yet been characterized in vivo, one could conclude that a virus that is unable to avoid RNA degradation by the host would likely be attenuated in animal models of infection.

In addition to the viral cis-features of the alphavirus 3′UTRs, there are instances whereby aspects of the host RNA regulatory pathways influence alphaviral pathogenesis. These include host derived MicroRNAs (miRNA) which are short host RNAs that regulate protein translation in a transcript specific manner. These regulatory RNAs can have both pro-viral and antiviral functions and can influence viral replication directly by targeting viral RNA or indirectly by targeting host mRNAs to alter the cellular environment. In situations where the miRNA directly interacts with viral RNA, viral replication is either restricted due to miRNA inhibition of viral genomic translation or enhanced due to the miRNA increasing the stability of the viral RNA [49,50,51].

**Figure 5:**
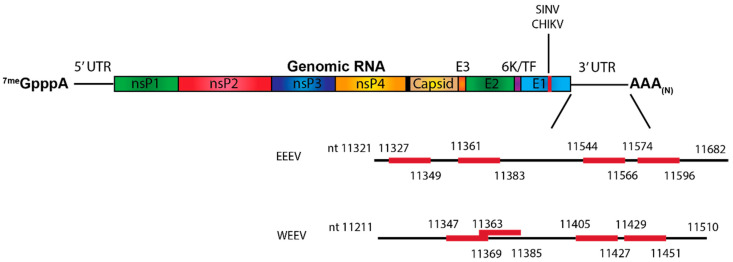
MicroRNA Binding Sites Known to Influence Alphaviral Pathogenesis. The miR-142-3p binding sites in the 3′ UTRs of EEV and WEEV as well as the miR-124 binding site in E1 of SINV and CHIKV are indicated in red [52,54].

The most well-described miRNA known to impact alphaviral pathogenesis is miR-142-3p. This hematopoietic-specific miRNA has been shown to bind to the 3′UTR of both EEEV and WEEV and restrict viral replication in myeloid-lineage cells (Figure 5) [49,52]. The restriction of myeloid cell replication leads to minimal type I IFN induction, allowing the virus to replicate and spread early during viral infection without activating a significant host antiviral response. This suppression of the innate immune response early during infection allows the virus to spread to the central nervous system (CNS), where severe encephalitis can then develop. Disruption of the miR142-3p binding sites resulted in the virus being able to replicate in myeloid cells but also resulted in significantly higher levels of type I IFN and a significant reduction in mortality in EEEV infected mice. The same has been found to be true for WEEV [52]. VEEV, however, does not contain any miR-142 binding sites, potentially explaining why VEEV is highly myeloid cell tropic whereas EEEV is not [52,53].

While most miRNA:vRNA interactions occur in the 5′ and 3′ UTRs, an interaction site in the structural open reading frame (ORF) of SINV and CHIKV has been recently described. Specifically, the interaction of the neuronal-specific miR-124 with the E1 region of SINV and CHIKV has been shown to positively regulate viral replication (Figure 5) [54]. Although this study did not specifically determine whether miR-124 was binding with the genomic RNA, the subgenomic RNA, or both of the vRNAs, miR-124 was shown to specifically increase subgenomic translation, while genomic translation remained unaffected. This suggests that miR-124 binds to the subgenomic RNA and not the genomic RNA, although the mechanism by which miR-124 differentiates between the two vRNA species is unknown. However, whether this interaction site is conserved in the encephalitic alphaviruses and how disrupting this interaction might affect pathogenesis are unknown.

### 4.3. Virulence Traits of the Nonstructural and Structural Open Reading Frames

The nonstructural and structural open reading frames comprise the vast majority of the genomic and subgenomic RNAs and the sequences of these regions are much less varied between the different alphaviruses, especially in comparison with the 5′ and 3′ UTRs. Relatively little is known about how the RNA structures, modifications, and RNA:Protein interactions that occur in these portions of the viral RNA contribute to pathogenesis outside of influencing the nonstructural and structural proteins they encode. Furthermore, whether there are differences between the structural open reading frames of the genome and subgenome that influence pathogenesis in different ways is also largely unknown. In spite of all that is unknown, there are a number of studies that illustrate several aspects of the viral genome and subgenome that are known to be important determinants for alphaviral pathogenesis.

#### 4.3.1. Dinucleotide Motif Biases in Alphaviral Pathogenesis

In studies assessing the usage of dinucleotide motifs and codon pairs in viruses relative to their hosts, it was found that many arboviruses utilize codon pairs which are overrepresented in their vertebrate hosts as opposed to the invertebrate vector [55]. Alphaviruses, however, do not utilize codon pairs that are overrepresented in either the vertebrate host or invertebrate vector species. Additionally, alphaviruses have a low codon bias and have roughly equivalent abundances of all dinucleotide motifs, with the UpG pair having a slightly higher abundance than the others [55,56,57]. This is rather unusual among ssRNA viruses, which typically suppress CpG and UpA dinucleotide pairs as they allow the viral genome to be targeted by antiviral proteins ZAP and RNaseL [57,58,59]. The lack of CpG suppression in alphaviral RNA leads them to be susceptible to ZAP and RNaseL antiviral activity leaving them subject to RNA degradation and inhibition of viral replication in tissue culture [60,61,62,63].

ZAP suppression of alphaviral replication is important for host type-I IFN mediated antiviral defense, as suckling mice deficient in ZAP are unable to adequately control SINV replication and have increased mortality [64]. In mice with mature immune systems, however, ZAP seems to contribute to pathogenesis, as 23-day old mice deficient in ZAP experience significantly decreased mortality in response to SINV infection. In the absence of ZAP, viral replication is increased in the peripheral tissues but decreased in the CNS. This is due to the increased viral replication in the peripheral tissues resulting in an increased innate immune response, resulting in a protective effect during viral infection of the CNS [64]. Therefore, by limiting viral replication early during infection, ZAP also limits the early host antiviral response which would otherwise prevent the virus from efficiently replicating in the CNS. This suggests that alphaviruses may have preserved CpG and UpA dinucleotide motifs as a means of using ZAP to evade the early immune response, and allows replication to high titer in the CNS.

#### 4.3.2. The Opal Stop Codon

**Figure 6:**
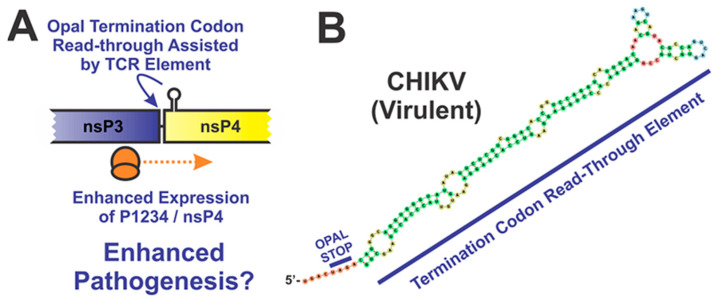
Importance of the Opal Stop Codon to Alphaviral Pathogenesis. (**A**) The Opal stop codon and the termination codon read-through (TCR) element present in nsP3/nsP4 are thought to play a role in controlling the expression of the nsP4 protein [68,70]. (**B**) The secondary structure of the TCR is found in CHIKV. The 5′ terminus is labeled, as is the Opal Stop codon which distinguishes the coding regions of nsP3 and nsP4. The TCR element is underlined.

A key feature of many alphaviruses is the presence of an Opal stop codon in between the nsP3 and nsP4 coding regions. Several studies have identified the Opal stop codon as a virulence determinant, but the overall impact of the Opal stop codon is, interestingly, highly dependent on the particular alphavirus species. Mutation of the Opal stop codon in CHIKV results in reduced pathogenicity, as mice infected with a virus where the Opal codon has been replaced with arginine experienced decreased footpad swelling and reduced inflammation in the joints [65]. In contrast, the introduction of the Opal stop codon in SFV, which naturally lacks a stop codon between nsP3 and nsP4, led to significant attenuation of disease in adult mice [66]. There is also further evidence that the importance of the Opal stop codon to virulence may be dependent on the host. In the O’nyong’nyong virus (ONNV), the presence of the Opal stop codon results in increased viral replication in BHK-21 cells but drastically decreased replication in C6/36 mosquito cells [67]. While the mechanism by which the Opal stop codon impacts virulence has not been characterized, it is thought to play a role in controlling the expression of the nsP4 protein (Figure 6) [68]. However, if this were universally true, then similar phenotypes would be anticipated across all alphavirus species. As this is not the case, it remains possible that alternative mechanisms contributing to pathogenesis exist.

In addition to the Opal stop codon, there is a proximal stem-loop structure that promotes read-through by the ribosome. Several studies have shown that the presence and stability of this stem-loop structure influences the expresion of nsP4, but the overall impact of this secondary structure motif on alphaviral pathogenesis is unclear as direct studies involving animal models have not been pursued [69,70]. Regardless, one could reasonably presume that deficits in the synthesis of the replication machinery would negatively impact the capacity of the virus to induce pathogenesis.

#### 4.3.3. Secondary Structures of the Alphaviruses Open Reading Frames

Due to their nature as single-stranded RNA viruses, the alphaviral RNAs are capable of folding into a diverse array of secondary structures during the viral lifecycle. Indeed, recent studies have shown the presence of extensive RNA secondary structures throughout the viral genome [71]. However, the specific functions of these secondary RNA structures and their importance to viral pathogenesis have not yet been characterized. In terms of the antiviral response, branched RNA structures are a known PAMP detected by MDA5, resulting in the induction of type-I IFN. Alphaviruses are known to activate MDA5, which, along with RIG-I, is thought to be one of the primary sensors of infection and inducers of type-I IFN [72,73]. CHIKV infected MAVS -/- mice, which prevents MDA5 from being able to induce an antiviral response through disruption of the innate signaling pathway, exhibit increased viremia and significantly decreased type-I IFN compared to CHIKV infected wild type mice [73]. Overall, this suggests that the general RNA secondary structure present in alphaviral RNA is important for determining the host antiviral response to infection.

In addition to the generalized structured nature of the alphaviral genome and the likely implications of such, there are several key secondary structures with specific identified roles during infection. These include the Packaging Signal (PS) located in the nonstructural ORF, and the aforementioned DLP element, and the 6K/TF Frameshift structures located in the structural ORF.

The primary role of the PS is to select the genomic RNA for encapsidation during viral infection. The precise location of the packaging signal varies across the members of the genus, with the majority of the alphaviruses having a packaging signal in nsP1, and SFV and RRV having their packaging signal in the coding region of the nsP2 protein [74,75]. The PS in nsP1 consists of 4 to 6 stem-loop RNA structures with conserved GGG sequences at the base of the loops [75]. Disrupting the stem-loop structures or mutating the GGG sequences results in the genome no longer being preferentially packaged, resulting in increased packaging of the subgenomic RNA, the most abundant RNA present during alphaviral infection. Packaging of the subgenome instead of the genome leads to decreased infectivity of viral particles, as the subgenome is not capable of initiating viral replication. This increase in the production of non-infectious particles would certainly negatively impact viral infection and pathogenesis in animal models, although the effect of mutating the packaging signal on alphaviral disease has not been specifically tested.

**Figure 7:**
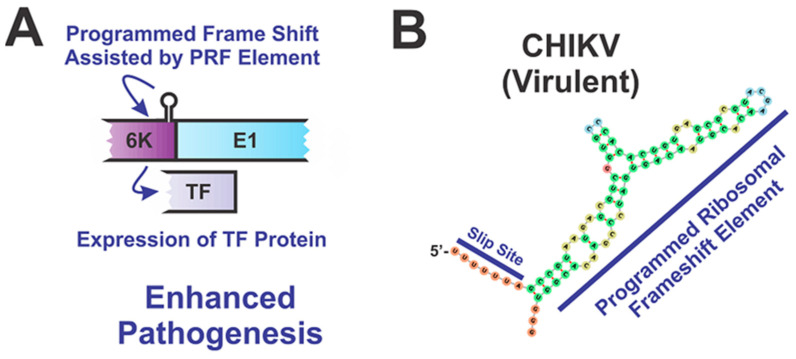
Impact of the 6K/TF Frameshift structure on Alphaviral Pathogenesis. (**A**) The presence of a frameshift slippage site followed by a series of tandem stem-loops (programmed frameshift element; PRF) allows for the production of the transframe (TF) protein [76]. Proper expression of the TF protein has been shown to be correlated with neuropathogenesis for multiple alphaviruses [77,78]. (**B**) The secondary structure of the PRF of CHIKV, with the site of the frame-shift indicated. Also labeled is the PRF element.

The second known structural element in the structural ORF of the subgenomic RNA that contributes to pathogenicity is the stem-loop which causes a frameshift during the translation of the 6K protein resulting in the production of the transframe (TF) protein (Figure 7) [76]. This element has been observed in SINV, VEEV, and CHIKV [70,77,78]. Disruption of the frameshift structure reduces the amount of TF produced, resulting in significantly decreasing infectious particle production as well as decreasing pathogenesis in SINV and VEEV infected mice [77,78]. More specifically, two-week-old CD-1 mice intracerebrally infected with SINV mutants with the frameshift structure disrupted experienced milder symptoms of the disease as well as significantly decreased mortality. Similarly, in VEEV, disruption of the frameshift structure resulted in decreased neuropathogenesis in six-week-old BALB/c mice [78]. Frameshifting efficiencies have been found to be different for each of the alphaviruses and are dependent on a set of tandem stem-loops downstream of the initial slippage site [78]. Whether disrupting the frameshift stem-loop will have the same impact on pathogenicity in the other alphaviruses as it did on SINV and VEEV pathogenesis has yet to be seen [79].

### 4.4. Protein:vRNA Interactions

While numerous viral and host protein interactions with the alphaviral RNA have been described, the best-characterized interactions are those that impact viral translation or vRNA synthesis. Interfering with the interactions would certainly affect alphaviral pathogenesis, as efficient translation and synthesis of the alphaviral RNA are vital for viral replication as well as for impairing the host immune response. However, because these interactions only seem to indirectly affect alphaviral pathogenesis because they deal with important aspects of the viral life cycle, they are beyond the scope of this review and will not be discussed in detail.

## 5. Contributions of Other Alphaviral RNAs to Pathogenesis

In addition to the two major positive-sense RNAs produced during alphaviral infection, there are several other RNA species with known and/or unknown functions that are produced at non-trivial amounts during infection. These include the negative-sense minus-strand RNA, the cryptic RNA II positive-sense RNA species, and defective viral RNAs. In this section, the known functions and contributions of each of these “other” viral RNA species are described.

### 5.1. The Contribution of the Minus-Strand RNA to Alphaviral Pathogenesis

Functionally, the minus-strand RNA serves as the template for the synthesis of all other positive-sense viral RNAs. It is a primary product of the viral replication cycle and is synthesized by the earliest forms of the alphaviral replication complex. Indeed, the maturation of the alphaviral nonstructural polyprotein rapidly diminishes the synthesis of the minus-strand RNA in favor of the two positive-sense RNA species.

Even though the function of the minus-strand RNA is believed to be well understood, the role of the minus-strand RNA in pathogenesis is less clearly defined. What few RNA structures and protein:RNA interactions have been described for the minus-strand RNA are in the context of viral replication and transcription. While disruption of features that promote efficient production of the viral genome and subgenome would have obvious impacts on the development of disease. As the mechanistic effects of these mutations involve fundamentally altering viral replication, they are beyond the scope of this review. Thus, the full extent of the RNA secondary structures present in the minus-strand RNA as well as any connected protein:RNA interactions and how they impact alphaviral pathogenesis has not yet been characterized. One aspect of the minus-strand RNA important to viral pathogenesis is that it can activate the various innate immune sensors which target non-self RNAs, such as RIG-I and MDA-5. To avoid being detected by these sensors, alphaviruses sequester the minus-strand RNA into replication spherules formed from host membranes [80]. However, isolation of the minus-strand RNA into replication spherules is thought in some cases to be incomplete, resulting in the activation of innate immunity and production of type-I IFN [16]. Therefore, how well the virus is able to isolate its minus-strand RNA from innate immune sensors likely plays a large role in determining pathogenicity. Whether there are RNA features that contribute to the formation of the spherules and the sequestration of the replication intermediates is unknown at this time.

### 5.2. The Alphaviral RNA II- a Consistent Curiosity

**Figure 8:**
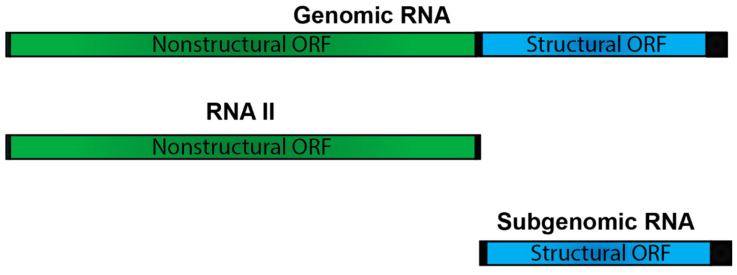
Positive-Sense Viral RNAs Produced During Infection. RNA II consists of the first ~7.6 kb of the genomic RNA, encompassing the nonstructural open reading frame [81]. The function of RNA II during alphaviral infection has not yet been well characterized.

The fourth viral RNA species that is generated during alphaviral infection is the positive-sense RNA species RNA II. RNA II consists of the first ~7.6kb of the genome and ends at the subgenomic promoter (Figure 8) [81]. RNA II is thought to be the single-stranded equivalent of replicative form II (RFII) which is generated during the synthesis of the subgenomic RNA. Although RNA II has been shown to be produced during replication of multiple alphaviruses, there is still very little known about it, such as whether or not it is capped and polyadenylated and whether it is packaged into viral particles [81,82,83,84]. It has been suggested that RNA II may potentially be involved in coordinating viral transcription and replication, but it may also bind a unique subset of proteins or RNAs and have functions separate from RNA synthesis [81]. Overall, while RNA II has been shown to be produced in significant quantity during typical alphaviral infection, little is known about its role in alphaviral replication and contribution to pathogenicity. Thus, renewed efforts designed to examine the impact of RNA II on infection are warranted.

### 5.3. Defective Viral RNA Species

Defective viral genomes (DVGs) consist of viral RNAs which have large deletions that render them unable to initiate infection. During viral replication, truncation, recombination, and/or rearrangement events of the viral genome can result in the production of DVGs. Alone, DVGs are unable to replicate as they lack a full complement of replication machinery and structural proteins. However, in the presence of the wild-type virus, the DVGs can be replicated and packaged into viral particles. During alphaviral infection, DVGs are typically produced during high MOI serial passaging studies and were found to repress viral replication [85,86]. In particular, the ability of DVGs to repress viral infection when present in excess has been shown for CHIKV, SFV, and SINV [87,88,89]. DVG production during in vivo infection has also been described for a number of alphaviruses, including CHIKV, SINV, VEEV, and Salmon Pancreatic Virus (SPV) [87,90,91,92,93]. DVGs are thought to impede viral infection by competing with full-length RNAs for viral replication complexes, leading to less efficient replication of the full-length viral RNA [86,94]. The antiviral activity of alphaviral DVGs in vitro is strain-specific, as DVGs produced by CHIKV do not exhibit the same antiviral effect on SINV and ONNV infection as they do during CHIKV infection [90].

Given the capacity for DVGs to restrict viral replication in tissue culture, there is the potential that DVG production could affect alphaviral pathogenesis in animal models as well. In SFV, mice inoculated intranasally with tissue culture-derived DVGs were protected from lethal infection, presumably because the DVGs interfered with viral dissemination and replication in the brain [95]. However, this was not found to be a universal trait of DVGs, as not all SFV derived DVGs were protective in mice, despite them showing antiviral activity in tissue culture [96]. Exactly what determines whether a particular DVG will be protective against lethal infection is largely unknown, although it does seem to be at least partially dependent on sequence and length [97,98].

Although the addition or excess production of DVGs during alphaviral infection is largely antiviral, the DVGs naturally produced over the course of infection may have different effects on viral pathogenesis. While the role DVGs play in the development of disease has not yet been shown for alphaviruses, it has been characterized for other RNA viruses. In the Sendai virus, DVGs have been shown to be immunostimulatory, as viral strains that produce higher levels of DVGs induce a greater IFN response than strains producing relatively few DVGs. In mice infected with the Sendai virus, DVGs produced by the virus have been found to be responsible for inducing the production of type-I IFN and triggering the host immune response [99]. In tissue culture, DVGs have been shown to promote persistence for a number of viruses, including Sendai, Ebola, West Nile, and Cytomegalovirus [100,101,102,103,104]. Therefore, it is possible that alphaviral DVGs may contribute to the high levels of inflammation seen during infection and may play a role in establishing persistent infection, although this has yet to be experimentally proven.

## 6. Conclusions and Future Perspectives

As summarized above, the alphaviral RNAs directly contribute to virulence and pathogenesis to a significant extent. These contributions are in addition to the obvious linkage between viral replication/RNA synthetic fitness and pathogenesis, and typically involve multiple aspects of the host/pathogen interface. The mechanisms by which the viral RNAs contribute to virulence are both direct, as in acting to directly evade or resist aspects of the host innate immune response, and indirect via the modulation of the production of viral proteins during infection. The alphaviral RNA virulence determinants are often, but not always, associated with secondary structures and may be found throughout the entire length of the viral RNA. Furthermore, virulence determinants that lack defined secondary structures often act as interaction sites for host and viral RNA-binding proteins. In these instances, the virulence determinant is directly linked to the primary sequences of the viral RNAs themselves.

Overall, the critical contributions of the alphaviral RNAs to pathogenesis have been established, but much work remains to identify the full extent to which the alphaviral RNAs are intertwined with pathogenesis and the precise mechanisms by which the viral RNA influence disease. While the knowledge compiled above represents the current state of understanding in this regard, it also highlights the presence of significant critical gaps in the understanding of the role of viral RNAs in pathogenesis.
